# Effect of water on the fluorine and chlorine partitioning behavior between olivine and silicate melt

**DOI:** 10.1007/s00410-017-1329-1

**Published:** 2017-03-16

**Authors:** Bastian Joachim, André Stechern, Thomas Ludwig, Jürgen Konzett, Alison Pawley, Lorraine Ruzié-Hamilton, Patricia L. Clay, Ray Burgess, Christopher J. Ballentine

**Affiliations:** 1 Institute for Mineralogy and Petrography, University of Innsbruck, Innrain 52, 6020 Innsbruck, Austria; 2 Department of Earth Sciences, University of Oxford, South Parks Road, Oxford, OX1 3 AN United Kingdom; 3 Institute for Mineralogy, Leibniz University of Hannover, Callinstrasse 3, 30167 Hannover, Germany; 4 Institute of Earth Sciences, Heidelberg University, In Neuenheimer Feld 234-236, 69120 Heidelberg, Germany; 5 School of Earth and Environmental Sciences, The University of Manchester, Manchester, M13 9PL United Kingdom

**Keywords:** Fluorine, Chlorine, Halogen, Partitioning, Water, H2O, Earth’s mantle, Mid Ocean Ridge Basalt (MORB), Ocean Island Basalt (OIB), Olivine, Forsterite, SIMS

## Abstract

Halogens show a range from moderate (F) to highly (Cl, Br, I) volatile and incompatible behavior, which makes them excellent tracers for volatile transport processes in the Earth’s mantle. Experimentally determined fluorine and chlorine partitioning data between mantle minerals and silicate melt enable us to estimate Mid Ocean Ridge Basalt (MORB) and Ocean Island Basalt (OIB) source region concentrations for these elements. This study investigates the effect of varying small amounts of water on the fluorine and chlorine partitioning behavior at 1280 °C and 0.3 GPa between olivine and silicate melt in the Fe-free CMAS+F–Cl–Br–I–H_2_O model system. Results show that, within the uncertainty of the analyses, water has no effect on the chlorine partitioning behavior for bulk water contents ranging from 0.03 (2) wt% H_2_O (D_Cl_^ol/melt^ = 1.6 ± 0.9 × 10^−4^) to 0.33 (6) wt% H_2_O (D_Cl_^ol/melt^ = 2.2 ± 1.1 × 10^−4^). Consequently, with the effect of pressure being negligible in the uppermost mantle (Joachim et al. Chem Geol 416:65–78, 2015), temperature is the only parameter that needs to be considered for the determination of chlorine partition coefficients between olivine and melt at least in the simplified iron-free CMAS+F–Cl–Br–I–H_2_O system. In contrast, the fluorine partition coefficient increases linearly in this range and may be described at 1280 °C and 0.3 GPa with (*R* ^2^ = 0.99): $D_{F}^{\text{ol/melt}}\ =\ 3.6\pm 0.4\ \times \ {{10}^{-3}}\ \times \ {{X}_{{{\text{H}}_{\text{2}}}\text{O}}}\left( \text{wt }\!\!\%\!\!\text{ } \right)\ +\ 6\ \pm \ 0.4\times \,{{10}^{-4}}$. The observed fluorine partitioning behavior supports the theory suggested by Crépisson et al. (Earth Planet Sci Lett 390:287–295, 2014) that fluorine and water are incorporated as clumped OH/F defects in the olivine structure. Results of this study further suggest that fluorine concentration estimates in OIB source regions are at least 10% lower than previously expected (Joachim et al. Chem Geol 416:65–78, 2015), implying that consideration of the effect of water on the fluorine partitioning behavior between Earth’s mantle minerals and silicate melt is vital for a correct estimation of fluorine abundances in OIB source regions. Estimates for MORB source fluorine concentrations as well as chlorine abundances in both mantle source regions are within uncertainty not affected by the presence of water.

## Introduction

Owing to their incompatibility and volatility, the distribution of H_2_O and halogens in the Earth’s mantle is influenced by processes such as fluid mobility, oxygen fugacity, fractionation, degassing, and partial melting. With quantification of their distribution between different mantle phases, this makes halogens excellent tracers of volatile transport processes (e.g., Schilling et al. 1980; Ito et al. 1983; Jambon et al. 1995). The volatile budget of the mantle profoundly affects, for instance, its viscosity, and therefore, the mode of mantle convection (e.g., Steinbach and Yuen 1995) responsible for heat transport and planetary cooling, implying that knowledge of the Earth’s mantle volatile distribution will provide insight into the history and evolution of our planet. The determination of halogen abundances in Mid Ocean Ridge Basalt (MORB) and Ocean Island Basalt (OIB) source regions allows us to quantify volatile concentrations in their respective source regions (e.g., Beyer et al. 2012; Joachim et al. 2015). Comparing OIB source halogen concentrations with primitive mantle estimates enables us to better understand and quantify any volatile transport processes during recycling of oceanic crust.

For a long time, the only available approach to estimate bulk halogen concentrations, such as fluorine and chlorine, in MORB and OIB mantle source region was based on the analysis of element ratios, such as F/P, F/Sr and F/Nd, or Cl/K and Cl/Nb obtained from natural samples that were used as a proxy (Schilling et al. 1980; Ito et al. 1983; Michael and Schilling 1989; Déruelle 1992; Jambon et al. 1995; McDonough and Sun 1995; Newsom 1995; Wedepohl 1995; Saal et al. 2002; Salters and Stracke 2004; Le Roux et al. 2006; Workman et al. 2006; Shaw et al. 2008; Pyle and Mather 2009; Palme and O’Neill 2014). MORB and OIB source region estimates show, to date, a significant uncertainty. For example, fluorine OIB source region concentration estimates range from 8 ppm (Beyer et al. 2012) to 55 ppm (Kovalenko et al. 2006), which covers a range from depleted to enriched abundances relative to primitive mantle estimates.

An independent approach is the experimental simulation of partial melting processes at the respective Earth’s mantle source region conditions. By combining experimentally determined partition coefficients with natural halogen concentrations in oceanic basalts, halogen source region concentrations can be estimated. Recent experimental studies determined fluorine and chlorine partitioning data at pressure and temperature (P–T) conditions relevant for MORB and OIB source mantle regions between olivine, pyroxene, and corresponding silicate melt (Hauri et al. 2006; O’Leary et al. 2010; Beyer et al. 2012, 2016; Dalou et al. 2012; Joachim et al. 2015; Rosenthal et al. 2015). Joachim et al. (2015) showed that fluorine partitioning into olivine increases at nominally dry conditions by about two orders of magnitude between 1350 and 1600 °C at pressures ranging from 1 to 2.3 GPa. The effect of pressure on fluorine partitioning between olivine and melt is negligible at least for pressures ranging between 1 bar-2.5 GPa (e.g., Beyer et al. 2012; Joachim et al. 2015). Fluorine partitioning data between olivine and silicate melt were determined by Hauri et al. (2006) and Dalou et al. (2014) with melt H_2_O contents of 1.7–25 wt% and in the P–T range 1185–1245 °C and 1–4 GPa. Their data plot approximately 0.5–1 orders of magnitude above the trend shown in Joachim et al. (2015) and are roughly in agreement with partition coefficients determined at higher temperatures ranging from 1345 to1400 °C and pressures ranging from 1 bar to 2.5 GPa in nominally dry CMAS+F and NCMAS+F systems (Beyer et al. 2012). A potential explanation for the different fluorine partitioning behavior in hydrous and nominally anhydrous systems is the presence of water, which may lead to an increase of fluorine partitioning into olivine.

Chlorine partitioning between olivine and silicate melt is hardly investigated experimentally. Joachim et al. (2015) showed that chlorine partition coefficients between olivine and silicate melt in a nominally dry CMAS+F-Cl-Br-I-system increases by approximately 1.5 orders of magnitude between 1350 and 1600 °C at pressures ranging from 1.0 to 2.3 GPa. This increase is approximately comparable to the temperature effect on the fluorine partitioning behavior in the same temperature range. One data point provided by Dalou et al. (2014) from an experiment performed at 1240 °C and 1.2 GPa in the presence of a melt water content of 2.6 wt% plots about one order of magnitude above the trend shown in Joachim et al. (2015). This may indicate that the presence of water affects the chlorine partitioning behavior between olivine and melt.

To the best of our knowledge, no study have been available so far investigating the effect of variable bulk water contents on the fluorine and chlorine partitioning behavior between olivine and melt at a constant temperature.

Halogens may be incorporated into the olivine structure as point or planar defects. One type of planar defects known are humite-type lamellae (Kitamura et al. 1987; Drury et al. 1991; Risold et al. 2001; Stalder and Ulmer 2001; Wirth et al. 2001; Hermann et al. 2007). Transmission electron microscope (TEM) investigations give no indication for their presence at MORB or OIB source P–T conditions (Beyer et al. 2012; Joachim et al. 2015). This has led to the conjecture that fluorine and chlorine are incorporated as point defects in the olivine lattice at Earth’s mantle conditions. Potential mechanism may involve [MgO_2_]^2−^ = [F_2_]^2−^ as suggested by Bernini et al. (2013), or the replacement of a [SiO_4_]^4−^ tetrahedron by a [F_4_]^4−^ quadruplet (Crépisson et al. 2014) as proposed for fluorine incorporation in calcic and magnesian garnets (Valley et al. 1983; Smyth et al. 1990; Visser 1993).

Recent halogen mantle source region estimates indicate that the MORB source region is degassed by 22–88% in fluorine and 22–99% in chlorine relative to the primitive mantle (Joachim et al. 2015). The OIB source region, on the other hand, has a chlorine content similar to that of the primitive mantle, but is enriched in fluorine by a factor of 1.4–4.2 relative to the primitive mantle (Joachim et al. 2015). This implies that most of the subducted chlorine is transported back to the surface by arc volcanism, whereas significant amounts of fluorine reach deeper portions of the Earth’s mantle through subduction of oceanic lithosphere (Straub and Layne 2003; Joachim et al. 2015).

However, to date, none of the available fluorine and chlorine source region estimates consider the potential effect of water on the halogen partitioning behavior between olivine and melt. Bulk water abundance estimates for source peridotite beneath normal ridges (N-MORB) range from 50 to 250 ppm H_2_O (Michael 1988, 1995; Danyushevsky et al. 2000; Dixon et al. 2002; Saal et al. 2002; Simons et al. 2002; Hirschmann 2006; Green et al. 2014) and from 200 to 1100 ppm for H_2_O concentrations in OIB source regions (e.g., Dixon et al. 1997, 2002; Jamtveit et al. 2001; Hauri 2002; Hirschmann 2006; Green et al. 2014). Moreover, nominally dry high-pressure (Piston and Multianvil) experiments that simulate partial melting processes in mantle source regions to determine halogen partition coefficients are not perfectly dry (e.g., Kovács et al.^ 2012^; Green et al. 2014). Minor amounts of volatiles might be introduced to the capsule as surface water adsorbed on to the grains of pre-dried powder. In addition, metal capsules are permeable to traces of water in high-pressure experimental assemblies at high P–T conditions (e.g., Patiño Douce and Beard 1994; Truckenbrodt and Johannes 1999; Kovács et al. 2012; Green et al. 2014), so that water stemming from the natural pressure medium (e.g., talc) may infiltrate the capsule during the experimental runs (Joachim et al. 2012). This implies that water may affect the halogen partitioning behavior between olivine and melt in MORB and OIB source regions as well as during “nominally dry” high-pressure experiments that simulate partial melting processes in these source regions.

In this study, we investigate the effect of water on the fluorine and chlorine partitioning behavior between olivine and silicate melt at 0.3 GPa and 1280 °C. This allows us to evaluate the effect of water on fluorine and chlorine source region estimates and the potential consequences for our understanding of the Earth’s mantle halogen and volatile cycle.

## Experimental methods

### Rationale

Partitioning experiments were performed in the Fe-free CaO–MgO–Al_2_O_3_–SiO_2_ (CMAS)+F–Cl–Br–I–H_2_O system (Table 1). Knowledge about the effect of water on the halogen partitioning behavior between olivine and melt is a prerequisite for experimental setups that are able to control oxygen fugacities, e.g., using a double capsule technique (e.g., Boettcher et al. 1973; Matjuschkin et al. 2015). Thus, knowledge about the effect of water will enable us in a next step to introduce Fe at controlled oxygen fugacities to the system.

**Table 1:** EMP analyses of starting material glasses and experimental samples. The “halogen-free” glass was used as starting material for the synthesis of three “CMAS_start” glasses, which contain various amounts of H_2_O (Table 2) and halogens. These were used as starting materials for the partitioning experiments. Values represent the average of ten single measurements in the starting materials and five single measurements in each phase of each partitioning experiment. Uncertainties are given as 2σ. (b.d. = below detection limit). Crystal/melt ratios were determined via image analyses of BSE images using the software “ImageJ”

Sample	SiO_2_		Al_2_O_3_		MgO		CaO	F	Cl		Total	
*Starting materials*												
Halogen-free	46.88 (51)		18.89 (33)		18.39 (26)		17.02 (31)	b.d	b.d		101.18 (95)	
CMAS_start_dry	46.19 (57)		18.70 (44)		18.24 (19)		17.46 (30)	0.23 (4)	0.86 (2)		100.82 (118)	
CMAS_start_05	45.74 (57)		18.56 (47)		17.87 (33)		17.36 (23)	0.27 (4)	0.58 (2)		99.80 (145)	

Sample		SiO_2_		Al_2_O_3_		MgO	CaO	F		Cl	Total	Crystal/melt
*Partitioning experiments*												
Melt												
CMAS_dry		45.94 (62)		17.67 (22)		13.16 (30)	21.84 (41)	0.74 (4)		2.24 (12)	101.56 (100)	
CMAS_05		45.80 (88)		19.79 (33)		13.57 (38)	19.37 (62)	0.38 (6)		0.76 (6)	99.67 (156)	
CMAS_2		45.03 (86)		19.80 (50)		14.16 (24)	18.75 (43)	0.30 (4)		0.39 (4)	98.43 (153)	
Olivine												
CMAS_dry		43.12 (68)		b.d		57.69 (108)	0.58 (14)	b.d		b.d	101.39 (109)	0.12 (4)
CMAS_05		42.84 (44)		b.d		57.71 (71)	0.46 (3)	b.d		b.d	101.01 (98)	0.15 (5)
CMAS_2		42.20 (41)		0.13 (10)		56.84 (85)	0.44 (8)	b.d		b.d	99.61 (127)	0.21 (4)
Anorthite												
CMAS_dry		43.67 (87)		35.69 (19)		0.28 (17)	19.84 (53)	b.d		b.d	99.84 (108)	0.34 (3)
CMAS_05		43.58 (50)		35.81 (30)		0.30 (5)	19.79 (44)	b.d		b.d	99.48 (104)	0.06 (6)
Clinopyroxene												
CMAS_dry		52.09 (65)		6.22 (27)		18.09 (117)	24.06 (53)	b.d		b.d	100.46 (114)	0.04 (2)

**Table 2:** Nominal water content of starting materials calculated from their initial weight; water content of glassy starting materials was determined using FTIR analysis (see text and Fig. 2 for details); water content of melt pools after partitioning experiments was determined using SIMS; bulk water contents after partitioning experiments were calculated assuming that the water content in crystalline phases is negligible compared to the water content of melt pools (see "Halogen incorporation mechanism in olivine" for details): H_2_O_bulk − after experiment_ = H_2_O_SIMS−melt pool_ × (1-(crystal/melt)_total_) with (crystal/melt)_total_ being the bulk sample crystal/melt ratio derived from Table 1. Uncertainties are given as 2σ

Sample	H_2_O (nominal; starting material; wt%)		H_2_O (FTIR; starting material; wt%)	H_2_O (SIMS; melt pool; wt%)	H_2_O (bulk – after experiment; wt%)
CMAS_dry	0	b.d		0.06 (2)	0.03 (2)
CMAS_05	0.05	0.23 (2)		0.33 (4)	0.26 (7)
CMAS_2	0.2	0.30 (3)		0.42 (4)	0.33 (6)

Defined amounts of water and halogens were added as H_2_O, CaF_2_, CaCl_2_, CaBr_2_, and CaI_2_ (Tables 1, 2). Experiments were performed in a two-step approach using an Internally Heated Pressure Vessel (IHPV). First, hydrous, halogen bearing silicate glasses were synthesized at 1450 °C and 0.2 GPa to be used as starting materials. This method allows the exact determination of starting material compositions using the electron microprobe (EMP) for major element concentrations and infrared (IR) spectroscopy for bulk H_2_O abundances. All experiments were then performed in the same run, i.e., at identical P–T conditions (1280 °C, 0.3 GPa) and with almost identical major element starting material composition (Table 1) but varying bulk water content (Table 2). Large crystals were obtained using the temperature oscillation technique (Erdmann and Koepke 2016). Back-scattered electron (BSE) images and EMP analyses were acquired to examine the phase assemblage and distribution in each sample. The water content in melt pools after the run as well as fluorine and chlorine concentrations in olivines and melt pools were determined using secondary ion mass spectrometry (SIMS). Fluorine and chlorine melt-pool concentrations were confirmed by the EMP analysis. This experimental procedure allows the determination of fluorine and chlorine partition coefficients between olivine and melt (Table 3) at bulk water contents ranging from 0.03 to 0.33 wt% (Table 2), and enables us to assess the effect of water on the partitioning behavior. Bromine and iodine abundances in olivines were below the detection limit of the methods and will be part of a subsequent contribution using a new analytical technique that will be based on the determination of halogen abundances by the analysis of noble gases produced by neutron irradiation (Johnson et al. 2000; Ruzié-Hamilton et al. 2015).

**Table 3:** Fluorine and chlorine concentrations in olivine and melt were determined using SIMS. Fluorine and chlorine partition coefficients between olivine and silicate melt were calculated as *D* ^ol/melt^ = *C*_crystal_/*C*_melt_. Determination of uncertainties is described in detail in "Addition of water to experimental charges during preparation of high P-T experiments"

Sample	F_ol_ (SIMS; ppm)	Cl_ol_ (SIMS; ppm)	F_melt_ (SIMS; wt%)	Cl_melt_ (SIMS; wt%)	D_F_^ol/melt^ (10^−4^)	D_Cl_^ol/melt^ (10^−4^)
CMAS_dry	5.4 (14)	3.6 (17)	0.83 (9)	2.21 (14)	6.6 (29)	1.6 (9)
CMAS_05	5.7 (26)	1.5 (10)	0.37 (1)	0.83 (1)	15.6 (73)	1.9 (13)
CMAS_2	4.4 (23)	1.2 (6)	0.25 (1)	0.51 (1)	17.3 (76)	2.2 (11)

### Starting materials

Starting materials were prepared from pure, analytical grade oxides, carbonates, and halides. First, dried (105 °C, 30 min) CaCO_3_, MgO, Al_2_O_3_, and SiO_2_ powders were thoroughly mixed in an agate mortar and heated to 1600 °C for 3 h in a 1 atm chamber furnace using a platinum crucible. 105 °C is not sufficient to remove magnesium hydroxide resulting in a small weight-in uncertainty. After quenching, the glass was ground, remixed, and reheated to 1600 °C for 3 h. The “halogen-free” starting material (Table 1) was split into three equal parts. Defined amounts of halides were added as CaF_2_, CaCl_2_, CaBr_2_, and CaI_2_ (Table 1) to each part. Glasses were reground, mixed to homogenize all components, dried at 105 °C for at least 30 min, and subsequently filled into annealed platinum capsules of 4.8 mm inner diameter, 5.2 mm outer diameter, and 4 cm length. Defined small amounts of water (0.05 wt; 0.2 wt%; Table 2) were added to two of the capsules using a micro syringe and the capsules were sealed shut immediately using a point welder. The third capsule was dried at 600 °C for 1 h without addition of H_2_O before it was subsequently sealed shut to provide as dry conditions as possible.

Synthesis of starting materials and partitioning experiments was performed in an IHPV at the Leibniz University of Hannover, Germany. A detailed description of this apparatus is given in Berndt et al. (2002). The capsule was placed close to the maximum temperature position in the sample holder between two furnace controlling thermocouples (S-Type). Temperature at the capsule position was controlled by a third thermocouple (S-Type). For synthesis of starting materials, capsules were heated to 1450 °C at a constant pressure of 0.2 GPa with argon gas used as pressure medium, and held for at least 14 h. To avoid the formation of quench crystals during cooling, capsules were quenched rapidly by melting a platinum wire that held the capsule in place, so that the capsule dropped to the cold zone of the sample holder (Berndt et al. 2002). Following quenching, small glass fragments were separated from both ends of the glass cylinder and double polished for EMP and FTIR analyses (see "Textural observations and major element compositions"). The remaining glasses were reground in an agate mortar, dried at 105 °C for at least 2 h, and used for subsequent partitioning experiments.

### Partitioning experiments

After a series of test experiments, 1280 °C and 0.3 GPa were chosen as ideal P–T condition for the execution of all partial melting experiments presented in this study. At this P–T condition, all samples reveal a mixture of large crystals embedded in silicate melt and a crystal/melt ratio of about 0.2–0.5 (Table 1). This enables us to analyze the fluorine and chlorine concentrations in olivine and melt and the water concentration in the melt of each sample (Tables 1, 2, 3).

**Fig. 1:**
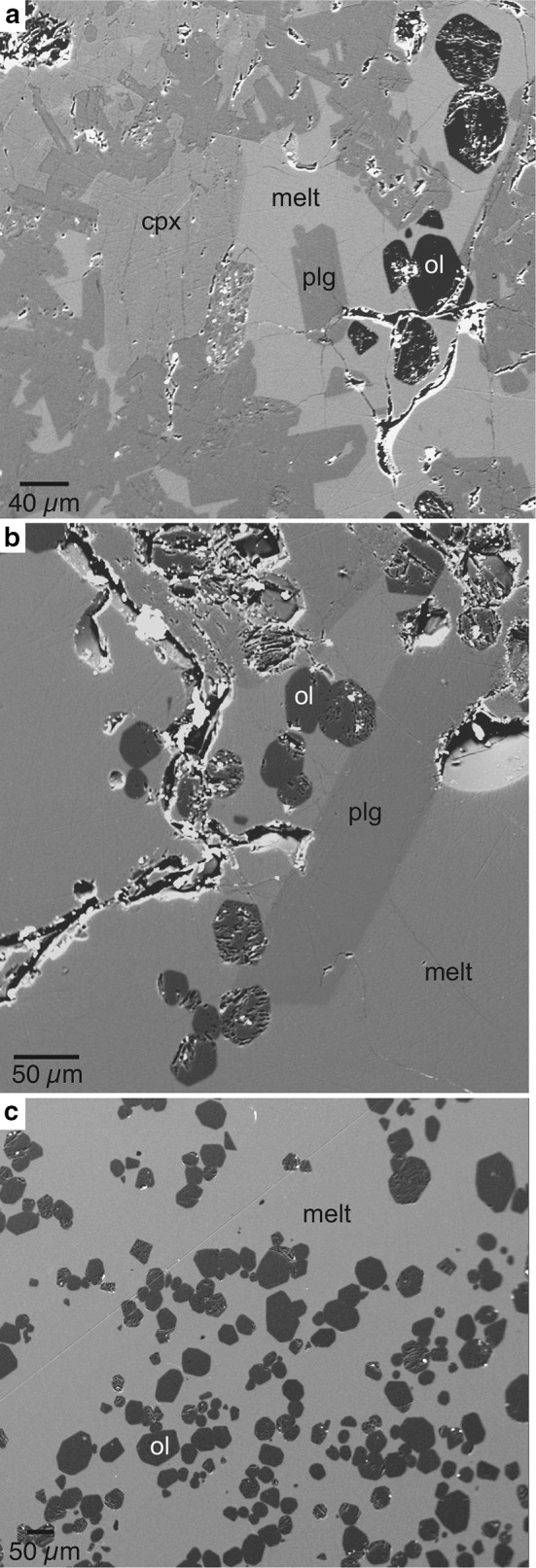
Back-scattered electron images of IHPV runs showing a silicate melt containing **a** clinopyroxene, plagioclase, and olivine (CMAS_dry), **b** plagioclase, olivine (CMAS_05), and **c** olivine (CMAS_2)

Dried starting materials (Table 1) were filled into annealed platinum capsules of 2.8 mm inner diameter and 3.2 mm outer diameter and 9 mm length, which were sealed shut immediately using a point welder. All experiments were performed in an IHPV located at the Leibniz University of Hannover. The three platinum capsules containing the three starting material compositions (Table 1) were placed in one sample holder, so that all three experiments with varying water contents were performed simultaneously in one run at identical pressure and temperature conditions. Pressure was kept constant at 300 ± 5 MPa throughout the run (pressure medium: dry argon gas). Experiments were first heated to the target temperature T_target_ = 1280 ± 2 °C at a rate of 50 °C/min and held for 1 h. Following this, the temperature was set to oscillate with a period of 1 h between T_target_ +20 °C and T_target_ -20 °C (Erdmann and Koepke 2016). After 48 temperature oscillation cycles (with a total duration of 48 h), the temperature was kept constant for another 48 h to ensure equilibrium conditions. This method allows growth of large crystals (Fig. 1), which is a prerequisite for SIMS analysis. At the end of the runs, we applied the rapid quench technique to avoid nucleation of quench crystals (Berndt et al. 2002).

Quenched samples were polished, cleaned with ethanol and water, and mounted in pure indium following Hauri et al. (2002).

### Analyses

#### Electron microprobe

Samples were analyzed using a Cameca SX 100 EMP located at the Leibniz University of Hannover, Germany. BSE images were used to determine grain sizes, textures, and crystal/melt ratios in each sample. Chemical compositions of crystals and melts were obtained by wavelength dispersive analysis. Operating conditions for all analyses of crystalline materials were 15 kV acceleration voltage, 15 nA beam current, 10 s counting time on the respective peak, and 10 s on background with focused beam. Glasses (starting materials and melt pools) were analyzed using a defocused beam (5 µm) and 4 nA beam current. Well-known standards were used for EMPA calibration (Mg on MgO; Si on wollastonite; Al on Al_2_O_3_; Ca on wollastonite; Cl on NaCl; F on SrF_2_). Ten single spots in the glass of each starting material were analyzed. In the run products, five spots were taken in each phase of each sample with varying distance to the crystal–melt interfaces to determine the major element (CaO, MgO, Al_2_O_3_, SiO_2_), fluorine, and chlorine concentrations (Table 1).

#### Fourier transformation infrared spectroscopy (FTIR)

**Fig. 2:**
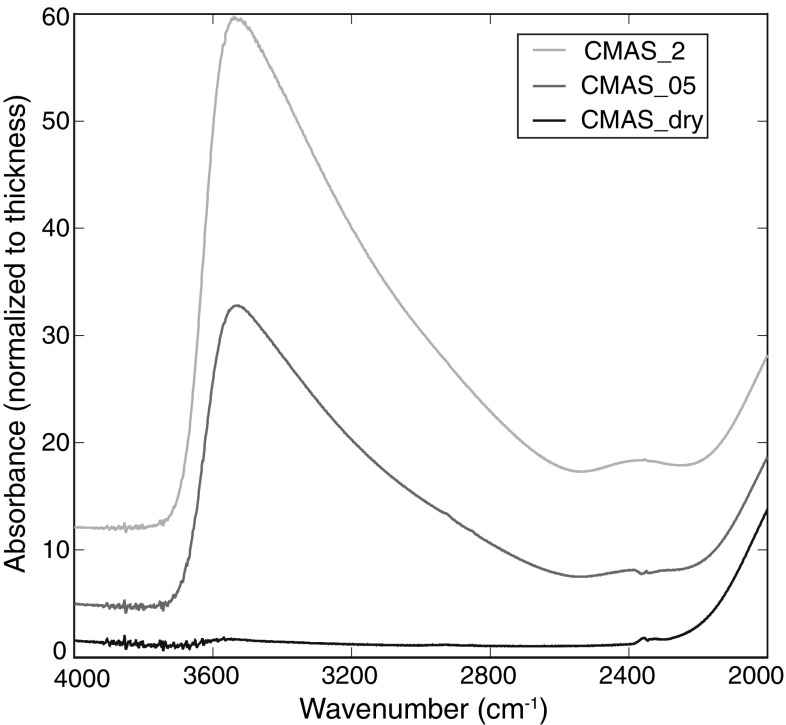
FTIR spectra of glassy starting materials “CMAS_start_dry”, “CMAS_start_05”, and “CMAS_start_2” showing the wavenumber region of the OH stretching vibration band at 3550 cm^−1^

Bulk water contents of starting materials were determined by Fourier Transformation Infrared Spectroscopy (FTIR). IR absorption spectra of doubly polished glass wafers with a thickness of 260–310 µm were recorded using a “Bruker IRscopeII” microscope connected to a “Bruker IFS88” FTIR spectrometer located at the University of Hannover, Germany. A Globar light source (MIR), KBr beam splitter, and a DTGS detector were used for each measurement. The measurement range was 2000–6000 cm^−1^. 50 single scans were averaged per spectrum with a resolution of 2 cm^−1^. A slit aperture was used between the objective and the detector to limit the analyzed sample volume. The area selected by the slit was about 100 × 100 µm wide. For each starting material, five individual measurements were taken at different positions and averaged. Spectra were fitted using the “OPUS” software. Starting material bulk water contents were quantified from the peak height of the OH stretching vibration band at 3550 cm^−1^ (Fig. 2) after subtracting a linear base line following the method of Mercier et al. (2010) and using their FTIR absorptivity coefficient for basalt glass. Densities of basaltic glasses could not be determined directly due to the small sample volume but were estimated to 2.6 ± 0.2 g/cm^3^ (e.g., Mercier et al. 2010). The starting material bulk water contents presented in Table 2 represent the average of five single analyses taken at different positions of each starting material.

#### Secondary ion mass spectrometry (SIMS)

##### Fluorine and chlorine concentrations in olivine and melt

Fluorine and chlorine concentrations of olivine crystals and glasses were determined in two separate sessions using a Cameca 1270 located at the University of Edinburgh, United Kingdom. First, samples were gold coated to provide electrical conductivity. Sample spots were pre-sputtered for 60 s at the beginning of each analysis to suppress surface contamination and amorphize the surface of crystalline materials. Pre-sputter and analytical conditions were identical. In total, in each sample, 10–30 single spots were analyzed in the olivines and 6–10 spots in the melt pools. Settings for all measurements are summarized as follows: A Cs^+^ primary ion beam with a primary current of 1 nA was accelerated to 10 keV and focused to a spot diameter of ~5 µm. Negative secondary ions were accelerated to 10 keV and analyzed using an electron multiplier detector with an energy width of 40 eV. Peak counting times were 4 s each for ^19^F and ^35^Cl, and 2 s for ^30^Si. Vacuum was set to 1·10^−7^ mbar. Mass resolving power was adjusted to M/ΔM ≈ 3000, which is adequate to resolve ^19^F from the potential interferences of ^18^OH and ^16^OH_3_. The typical challenging interference for the analysis of Cl is the SH molecule, which is not relevant in this study, because the system is sulfur free. Detection limits were about one ppm for both, fluorine and chlorine.

Fluorine and chlorine concentrations in minerals and melt pools were normalized to the ^30^Si count rate and determined using the fluorine (3000 ppm) and chlorine (7400 ppm) concentration of the “Halogen standard 3” reference material (Joachim et al. 2015). To verify the accuracy of this method, a BCR-2 g glass standard reference material (440 ppm fluorine; USGS certificate of analysis) and Lipari obsidian standard glass (0.36 wt% chlorine; Hunt and Hill 1993; Kuehn et al. 2009) were analyzed before the measurement of each sample. Melt-pool fluorine and chlorine concentrations were also determined using EMP analysis (Table 1). These provide a first-order independent method for the determination of F and Cl melt-pool abundances. Both methods give within uncertainty identical fluorine and chlorine concentrations (Tables 1, 3), confirming that SIMS analyses are within uncertainty accurate (Table 3 and "Addition of water to experimental charges during preparation of high P-T experiments").

The amorphous structure and composition of basaltic glasses (melt pools) is directly comparable to the composition and structure of the basalt glass “Halogen standard 3” (Joachim et al. 2015), so that, for this phase, only the uncertainty of the analytical method (SIMS) and the reproducibility of the measurements were considered for the determination of total analytical uncertainties. The pre-sputter process amorphizes crystalline materials, so that relative sensitivity factors of glassy standard materials can likely be applied to crystalline and amorphous materials (e.g., Stephan and Lyon 2013). A detailed study, which compares matrix effects of glass standards with defined halogen concentrations used for the analyses of crystalline samples, is, however, still missing. To make sure that the potential for a systematic error stemming from this matrix effect is adequately covered by the total uncertainty, we applied an additional conservative uncertainty of a factor of two for the determination of fluorine and chlorine abundances in olivines, following Joachim et al. (2015).

##### H_2_O melt-pool concentration

Hydrogen concentrations in the melt pools were determined with a Cameca ims3f ion probe at Heidelberg University using a ^16^O^−^ primary ion beam with a net energy of 14.5 keV, a beam current of 10 nA and a spot diameter of ~15 µm. Positive secondary ions were accelerated to 4.5 keV with an offset of 75 eV (energy filtering), an energy window width of 40 eV, and a mass resolving power of M/ΔM ≈ 400. To minimize *in situ* hydrogen contamination, the area analyzed was limited to ~6 µm (nominal imaged field 25 µm; field aperture 400 µm) and a liquid nitrogen cold trap in the sample chamber was used. Prior to 6 analysis cycles with an integration time of 3 s for H^+^ and ^30^Si^+^ per cycle, the sample was pre-sputtered for 240 s to remove the initial water and hydrocarbon surface contamination. The H^+^ background signal due to *in situ* contamination was equivalent to ≤50 µg/g H_2_O and at least a factor 10 below the lowest value analyzed (CMAS_dry: 0.06 wt%, Table 2). At a sensitivity of ~1 cps H^+^ for 1 µg/g H_2_O, the detection limit of this analytical setup was dominated by the intensity of the background signal. Because of the high H_2_O concentrations, it was not necessary to determine (and correct for) the rate of in situ contamination (see Ludwig and Stalder 2007 for a more detailed description of the setup). Quantification was done using relative ion yields (reference isotope ^30^Si) with a hydrated glass (5.9 wt% H_2_O, equivalent to glass ‘CG 1’ in Acosta-Vigil et al. 2006) as reference material.

## Results

### Textural observations and major element compositions

EMPA results show that all starting materials are homogeneous glasses and have within uncertainty identical major element concentrations (Table 1). FTIR results show that the bulk water content of the dry starting material is below the detection limit of the method (<0.1 wt%, Mercier et al. 2010). Glassy starting materials for experiments CMAS_05 and CMAS_2 show a bulk water concentration that is about 0.1–0.18 wt% higher than the bulk water concentration expected from their nominal weight (Table 2). After the run, melt-pool concentrations show a slight elevation of the bulk water content of 0.06–0.12 wt% compared to bulk water abundances in the respective starting materials before the partitioning experiment (Table 2).

EMP analyses reveal that olivines are forsterites with a small amount of Ca substituting for Mg in the crystal lattice (Table 1); plagioclases are anorthite and pyroxenes are clinopyroxene. Crystals in all experiments appear homogeneous (Fig. 1). EMP analyses at five different spots in each crystalline phase show little variation (Table 1), indicating that crystals do not have any major element concentration gradients. The three experiments vary significantly regarding their phase assemblage and crystal/melt ratio (Table 1). The nominally dry experiment “CMAS_dry” shows a mixture of olivine, plagioclase and pyroxene with a crystal/melt ratio of 0.48 (3) (Fig. 1a). BSE images of this experiment reveal that all crystals are predominantly euhedral and of rectangular shape. A few plagioclases show voids. Olivines and plagioclases have a side length of 30–60 µm; pyroxenes are larger and have a side length of up to 120 µm (Fig. 1a).

Sample “CMAS_05” shows a significantly lower crystal/melt ratio of 0.21(6). Observed crystals are predominantly euhedral olivines of rectangular shape with a side length of about 40 µm (Fig. 1b). The sample also shows a few isolated, euhedral plagioclase crystals of rectangular shape that reach a side length of more than 150 µm (Fig. 1b). In contrast to sample “CMAS_dry”, no pyroxene was observed in sample “CMAS_05”.

Sample “CMAS_2” shows euhedral olivines, which are identical in shape, size, distribution, and major element composition to the ones observed in sample “CMAS_05” (Table 1; Fig. 1c). The crystal/melt ratio of sample “CMAS_2” (0.21(4)) is within uncertainty also identical to the ratio in sample “CMAS_05”. However, olivine is the only crystalline material in sample “CMAS_2”; neither plagioclase nor pyroxene was observed. Melt pools in all samples appear homogeneous.

EMP analyses taken at five different spots in the melt pools of each sample show only little variation (Table 1) and give no indication for a depletion or enrichment of any element adjacent to the crystals. Variations in melt-pool compositions between different experiments are dependent on the crystal/melt ratio and the observed phase assemblage.

### Addition of water to experimental charges during preparation of high P–T experiments

Halogen-free melt was produced by heating a homogeneous halogen-free powder mix to 1600 °C in an open Pt crucible. Due to the high temperature, we may certainly assume that the halogen-free glass is almost completely dry. Water and halogen bearing glassy starting materials show actual bulk water concentrations determined using FTIR analyses (Table 2) that are 0.1–0.18 wt% higher than expected from their initial weight. The difference may be explained by the fact that adsorbed surface water is introduced in significant amounts to the assembly during capsule preparation mainly through strongly hygroscopic calcium-halide powders. Usage of dried starting materials (at 105 °C) and subsequent welding of the filled capsule is not sufficient to avoid the addition of surface water. IR spectra of the halogen bearing dry starting material (CMAS_dry) reveal that the bulk water content of this sample is lower than the detection limit of this method (<0.1 wt%, Mercier et al. 2010). In contrast to the water bearing starting materials, this starting material was dried at 600 °C and welded shut within 60 s after removing it from the furnace, because no addition of water was required.

Melt-pool water contents after partial melting experiments were determined using SIMS. As a first order approximation, it is assumed that water behaves almost perfectly incompatibly and is completely distributed into the melt pools. Considering crystal/melt ratios, bulk sample water contents after the experiments were calculated according to H_2_O_bulk − after experiment_ = H_2_O_SIMS−melt pool_ × (1-(crystal/melt)_total_) with (crystal/melt)_total_ being the bulk sample crystal/melt ratios (Table 1). Bulk sample water contents are within uncertainty identical to starting material bulk water concentrations determined using FTIR (Table 2). The general trend seems to indicate the addition of very small amounts of water to the experimental charges through adsorbed surface water, because average bulk H_2_O concentrations are 0.03–0.04 wt% higher compared to starting material bulk H_2_O concentrations. However, addition of such small amounts is within the uncertainty of the analysis.

### Effect of water on fluorine and chlorine partitioning behavior between olivine and melt

We considered only monocrystalline, large, euhedral olivines for SIMS analyses. Nevertheless, a few single analyses of olivine crystals show ^19^F/^30^Si and ^35^Cl/^30^Si ratios that are an order of magnitude or more above the respective average ratio. It cannot be excluded that small melt fragments are part of the ablated material. Owing to the incompatible behavior of fluorine and chlorine, even the ablation of very small glass fragments causes a significant increase of the respective halogen/^30^Si ratio. Single measurements of olivine that show a significant increase of an order of magnitude or more in both, the ^19^F/^30^Si and ^35^Cl/^30^Si ratio, are therefore, interpreted as being affected by glass fragments and were not considered further.

Fluorine and chlorine partition coefficients $(D_{X}^{\text{olivine/melt}})$ were calculated from the halogen concentrations in olivine $(C_{X}^{\text{olivine/melt}})$ and corresponding quenched melt $(C_{X}^{\text{melt}})$ according to $D_{X}^{\text{olivine/melt}}=C_{X}^{\text{olivine}}/C_{X}^{\text{melt}}$ (Table 3). In contrast to absolute fluorine and chlorine concentrations, determination of partition coefficients is not dependent on the quality of standard materials as potential inaccuracies would affect the determination of absolute halogen concentrations in both, olivine and glass, in an identical manner, if we exclude a potential matrix effect.

The fluorine partitioning behavior between olivine and melt increases linearly with increasing water content (bulk sample water content: 0.03(2)–0.33(6) wt% H_2_O; see Table 3 and "Halogen incorporation mechanism in olivine' for details) and may be described at 1280 °C and 0.3 GPa with (*R* ^2^ = 0.99):

1 $$~~~D_{F}^{\text{ol/melt}}=3.6\pm 0.4\ \times \ {{10}^{-3}}\ \times \ {{X}_{{{\text{H}}_{\text{2}}}\text{O}}}+6\pm 0.4\ \times \ {{10}^{-4}}$$

with X_H2O_ being the bulk sample water content in wt%. Uncertainties are given as 1σ. The slope of this function is not affected by a potential matrix effect, because this would be identical in all analyzed samples.

**Fig. 3:**
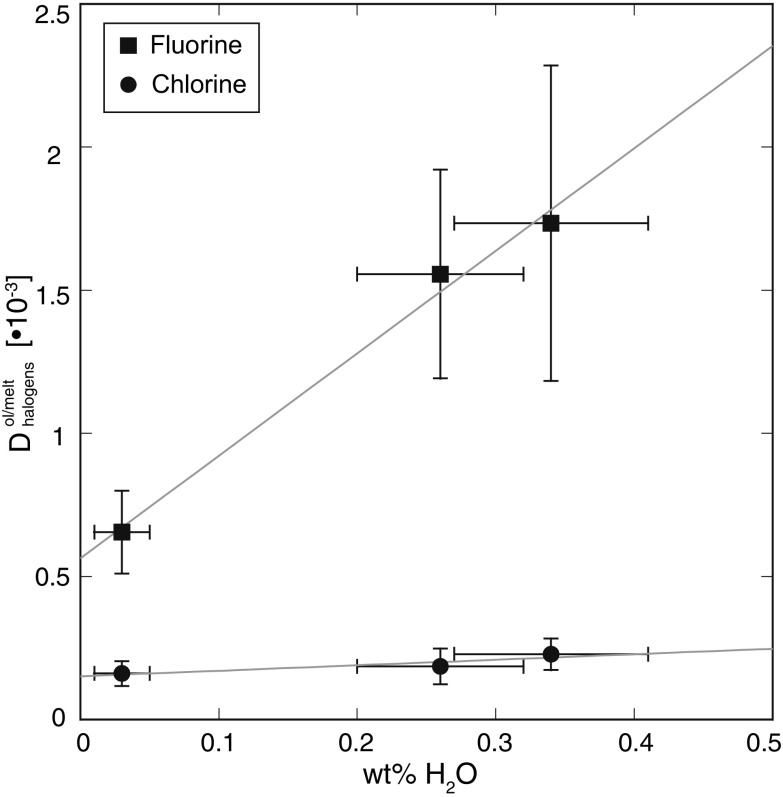
Plot of fluorine and chlorine partition coefficients between olivine and silicate melt (Table 3) vs. the sample bulk water content after partitioning experiments (Table 2). All experiments were performed in one run, i.e., at identical P–T conditions of 1280 °C and 0.3 GPa. Fluorine partition coefficients increase linearly with increasing bulk water content, whereas the chlorine partitioning behavior is, within uncertainty, not affected by the presence of water between bulk water contents of 0.03 (2) and 0.33 (6) wt% H_2_O

The presence of water has, within uncertainty, no effect on the chlorine partitioning behavior between olivine and melt at 0.3 GPa and 1280 °C (Fig. 3).

## Discussion

### Halogen incorporation mechanism in olivine

The experiments presented in this study were not specifically designed to determine the fluorine and chlorine incorporation mechanism in olivine. The observed partitioning behavior gives, however, strong indications regarding the fluorine incorporation mechanism. A potential mechanism is the [MgO_2_]^2−^ = [F_2_]^2−^ substitution (Bernini et al. 2013), an analog mechanism of OH incorporation in olivine, in which protonation of oxygen coupled with the formation of Mg vacancies in the octahedral site is the predominant mechanism (Smyth et al. 2006).

**Fig. 4:**
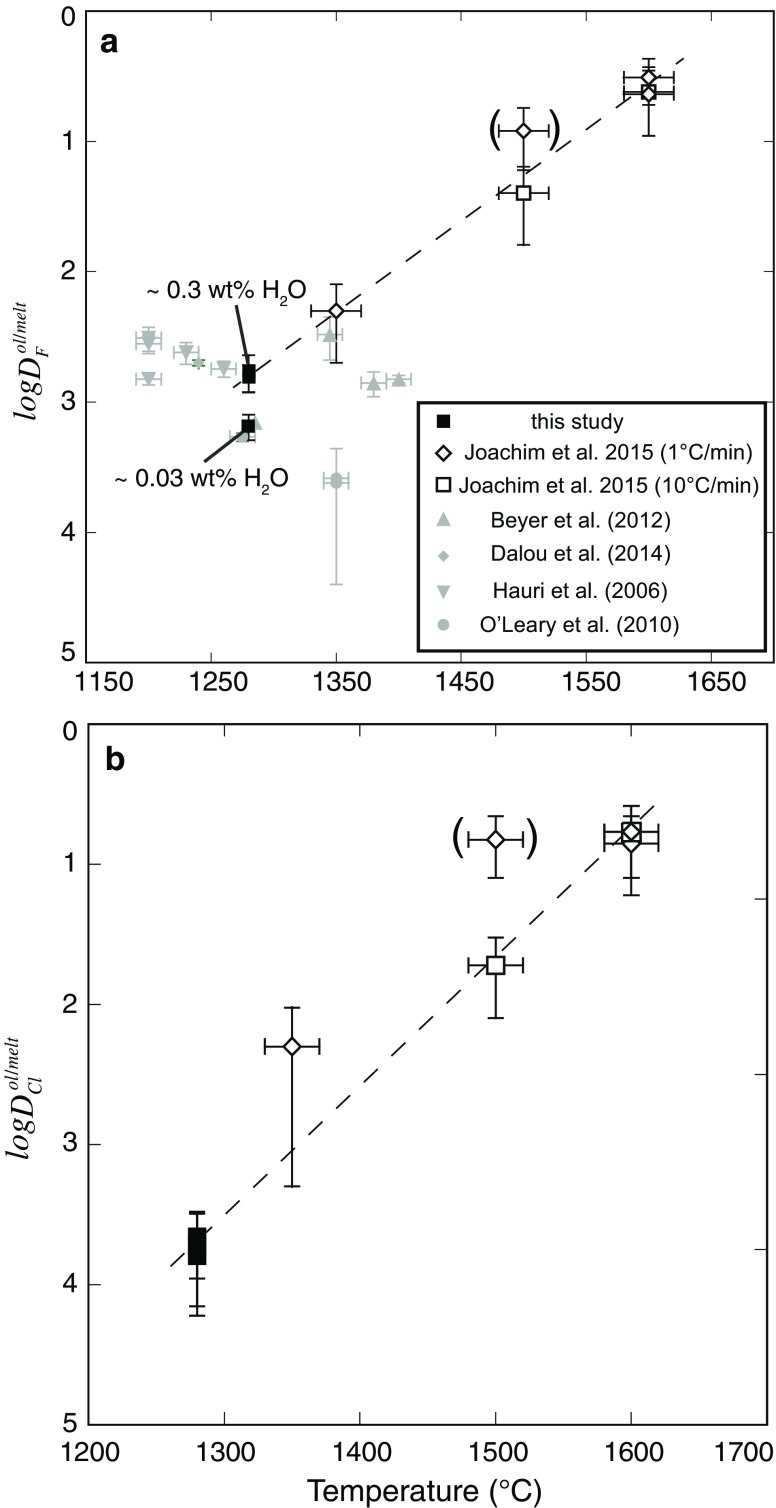
Plots of temperature vs. **a** fluorine and **b** chlorine partition coefficients between olivine and silicate melt. *Dashed lines* represent the best fit through the data of Joachim et al. (2015) and this study excluding the overestimated slow-cooled sample targeting at 1500 °C (cooled to target temperature with 1 °C/min; in *parentheses*; see Joachim et al. 2015 for details) and in (**a**) the almost dry sample (CMAS_dry; 0.03 (2) wt% H_2_O) of this study. A detailed discussion is provided in "Potential effect of water on fluorine concentrations and F/H_2_O ratios during partial melting in the upper mantle". Note that experiments of Joachim et al. (2015) and this study were performed between 0.3 and 2.3 GPa, implying that the effect of pressure is negligible in this range. The following equations describe the, **a** fluorine partitioning behavior between olivine and silicate melt in the presence of a bulk water content of approximately 0.2–0.3 wt% H_2_O and **b** chlorine partitioning behavior that is not affected by the bulk water content: **a** $D_{\text{ol}/\text{melt}}^{\text{F}}$ = 3E – 12e^0.0157 × T(°C)^ (*R* ^2^ = 0.99); **b** $\text{D}_{\text{ol}/\text{melt}}^{\text{Cl}}$ = 4E-16e^0.0211 × T(°C)^ (*R* ^2^ = 0.99)

A second potential mechanism is the replacement of a [SiO_4_]^4−^ tetrahedron by a [F_4_]^4−^ quadruplet, as proposed for fluorine incorporation in calcic and magnesian garnets (Valley et al. 1983; Smyth et al. 1990; Visser 1993). Using first-principle calculation, Bernini (2011) showed that this substitution mechanism increases the solubility of fluorine in forsterite exponentially with increasing temperature, whereas the effect of pressure is almost negligible. Bernini (2011) did not consider a potential effect of pressure on the incorporation mechanism of fluorine into olivine in dependence of varying bulk H_2_O. However, the solubility behavior is in agreement with the observed temperature dependent fluorine partitioning behavior described in Joachim et al. (2015; see also Fig. 4a of this study). Xue et al. (2016) have used ^1^H NMR measurements and first-principles calculations to show that the dominant H substitution mechanism in forsterite at 1200 °C and 12 GPa is [Si]^4+^ = [H]^4+^.

Based on FTIR analyses of water and fluorine bearing olivine crystals, Crépisson et al. (2014) suggested a coupled incorporation mechanism of fluorine and hydrogen in forsterite. In the presence of water, clumped OH/F defects are formed that are coupled with the formation of a [Si]^4+^ vacancy. Results of their study show that clumped OH/F defects increase the defect stability compared to solely hydrolytic weakening (Brodholt and Refson 2000), so that the combined incorporation of fluorine and hydrogen in the olivine structure increases the partitioning of fluorine into olivine. This behavior is in excellent agreement with results of this study, which show that fluorine partitioning into olivine increases linearly with increasing bulk water content. This strongly implies that [SiO_4_]^4−^ = [(F + OH)_4_]^4−^ is the dominant substitution mechanism for fluorine and water incorporation in olivine. However, it should be noted that this study as well as the study of Crépisson et al. (2015) was performed in a Fe-free model system. Future studies are strongly required that investigate the potential effect of Fe (and oxygen fugacity) on the fluorine incorporation mechanism in olivine.

In contrast to fluorine, within uncertainty, there is no effect of the bulk water content on chlorine partitioning behavior. An explanation for the different behavior might be that Cl is incorporated via another mechanism in the olivine structure, such as [MgO_2_]^2−^ = [Cl_2_]^2−^. However, specifically designed experiments combined with structural refinement methods (such as NMR spectroscopy) are required for a better understanding of the exact incorporation mechanism.

### Effect of water on estimates of fluorine and chlorine OIB source region concentrations

#### Chlorine

Results of this study show that within uncertainty, in the model system CMAS + F + Cl + Br + I + H_2_O, there is no effect of the bulk water content on the chlorine partitioning behavior between olivine and melt at 1280 °C, 0.3 GPa and bulk water contents ranging from 0.03 (2)–0.33 (6) wt%. Figure 4b shows that the chlorine partition coefficients determined in this study are perfectly in line with the temperature dependence of the chlorine partitioning behavior presented in Joachim et al. (2015). The data suggest that chlorine partitioning into olivine increases by three orders of magnitude between 1250 and 1600 °C (Fig. 4b), thus covering the complete temperature range relevant for partial melting processes in MORB and OIB source regions. Joachim et al. (2015) showed that the effect of pressure is negligible between 1.0 and 2.3 GPa. The fact that derived partition coefficients of this study are perfectly in line with the results of Joachim et al. (2015) implies that the effect of pressure is negligible down to at least 0.3 GPa. Consequently, at least in the simplified Fe-free CMAS + F-Cl-Br-I-H_2_O system, temperature is the only parameter that needs to be considered for the determination of chlorine partition coefficients between olivine and melt at uppermost mantle conditions.

#### Fluorine

In contrast to chlorine, the bulk water content does affect the fluorine partitioning behavior between olivine and silicate melt in the Fe-free model system CMAS + F + Cl + Br + I + H_2_O (Figs. 3, 4a) and thus potentially the estimates for fluorine concentrations in MORB and OIB source regions.

Figure 4a shows that fluorine partition coefficients for a bulk water content of 0.26 (7) −0.33 (6) wt% H_2_O (CMAS_05 and CMAS_2; Table 2) are perfectly in line with the temperature dependence of the fluorine partitioning behavior between olivine and melt presented in Joachim et al. (2015). The partition coefficient of the slow-cooled sample at 1500 °C (in parentheses in Fig. 4a) does not represent equilibrium conditions, and is, therefore, excluded from the fit (*see* Joachim et al. (2015) *for a detailed explanation*). The data point at almost dry conditions (0.03 (2) wt% bulk H_2_O), however, does not fit with the temperature trend presented in Joachim et al. (2015). This indicates that nominally dry piston-cylinder experiments (Joachim et al. 2015) were not dry but had a bulk water content at a level comparable to the water-doped experiments (~0.2–0.3 wt% bulk H_2_O) presented in this study (Table 1; Fig. 4a). The preparation procedures for halogen-doped glass powder starting materials used in Joachim et al. (2015) and the water-doped experiments presented in this study (CMAS_05 and CMAS_2, Table 2) were almost identical, implying that about 0.1 wt% bulk H_2_O was likely introduced as surface water to the experimental charges. With Pt capsules being permeable to traces of water in piston-cylinder assemblies at high P–T conditions (Patino Douce and Beard 1994; Truckenbrodt and Johannes 1999; Joachim et al. 2012), additional water present in the samples of the Joachim et al. (2015) study potentially infiltrated from the hydrous pressure medium (talc) into the capsule during the run resulting in a bulk water content of about 0.2–0.3 wt% H_2_O. Fluorine partitioning data provided by Hauri et al. (2006) and Dalou et al. (2014) are about 0.5 orders of magnitude above the trend shown in Joachim et al. (2015). This might be explained by the fact that these samples had a melt water content of 1.7–25 and 2.6 wt%, respectively, which would further increase fluorine partitioning into olivine.

Based on the results of this study, we are able to estimate, for the first time, the order of magnitude of the effect of water on fluorine mantle source region estimates. To this end, several assumptions have been made:

- We assume that the fluorine partition coefficient between olivine and silicate melt increases linearly with increasing bulk water content with the observed slope (Fig. 3) independent of pressure and temperature variations, i.e., that the observed effect of water on the fluorine partitioning behavior is valid at P–T conditions representing partial melting processes in MORB and OIB source regions.
- We assume that the piston-cylinder experiments presented in Joachim et al. (2015) contain a bulk water content of about 0.3 wt% H_2_O (Fig. 4a).
- We consider only the effect of water on the fluorine partitioning behavior between olivine and melt. Further studies are required to confirm that there is no potential effect of water on the partitioning behavior between other major mantle mineral phases and melt.
- We use the model of accumulated fractional melting developed by Shaw (1970) to estimate fluorine concentrations in MORB and OIB source regions. We are aware of the fact that this is a simplified model that does not consider incongruent melting of the Earth’s mantle. However, this reference model has also been used by Beyer et al. (2012) and Joachim et al. (2015) and allows direct comparison of our results with results of these studies. Furthermore, due to the simplifications of available experimental data (Fe-free CMAS system used as mantle analog; unknown effect of other volatiles, such as CO_2,_ on the water activity and thus halogen partitioning; analytical uncertainties particularly in determining halogen concentrations in olivine using SIMS (matrix effect); and potential effect of water on fluorine partitioning between other mantle minerals, such as opx, cpx or garnet, and melt), use of a more sophisticated model would at this stage not improve the quality of absolute source region estimates.

MORB and OIB source region fluorine estimates were calculated as described in Joachim et al. (2015), with identical input parameters except the consideration of the effect of water on the fluorine bulk partitioning behavior between olivine and melt. This allows us to directly compare the estimated fluorine source region concentrations of this study with estimates presented in Joachim et al. (2015).

In MORB source regions, H_2_O bulk concentrations ranging from 50 to 250 ppm are based on determinations of H_2_O/Ce ratios, H_2_O bulk analyses, and an experimental approach (e.g., Michael 1988, 1995; Danyushevsky et al. 2000; Dixon et al. 2002; Saal et al. 2002; Simons et al. 2002; Hirschmann 2006; Green et al. 2014). If we consider a minimum H_2_O bulk concentration of 50 ppm in MORB source regions, the bulk fluorine partition coefficient is estimated to be ${\stackrel{-}{\mathrm{D}}}_{\mathrm{F}}\left(\mathrm{M}\mathrm{O}\mathrm{R}\mathrm{B}\mathrm{ }\mathrm{s}\mathrm{o}\mathrm{u}\mathrm{r}\mathrm{c}\mathrm{e}\right)$ ≈ 0.004, which is only marginally lower than the estimate of 0.005 given in Joachim et al. (2015). Using this new average bulk partition coefficient, the MORB source region bulk fluorine concentration is calculated to be 3–14 ppm. There is no significant difference between this estimate and the estimate presented in Joachim et al. (2015), indicating that the effect of water on the fluorine partitioning behavior between olivine and melt is negligible for the determination of fluorine MORB source region concentrations. If we consider a higher bulk water concentration in MORB source regions, this would result in an even smaller effect of water on the bulk fluorine partition coefficient, because experimental samples of Joachim et al. (2015) contained a bulk water content of about 0.2–0.3 wt% H_2_O (Fig. 4a).

Geochemical studies of OIBs and oceanic plateaus (Dixon et al. 1997, 2002; Jamtveit et al. 2001; Hauri 2002; Nichols et al. 2002; Wallace et al. 2002; Seaman et al. 2004) of MORB that incorporate a plume-associated source component (Dixon et al. 2002) and an experimental approach (Green et al. 2014) suggest H_2_O concentrations in plume sources ranging from 200 to 1100 ppm. If we consider a minimum H_2_O bulk concentration of 200 ppm H_2_O, the bulk fluorine partition coefficient is estimated to be ${\stackrel{-}{\mathrm{D}}}_{\mathrm{F}}\left(\mathrm{O}\mathrm{I}\mathrm{B}\mathrm{ }\mathrm{s}\mathrm{o}\mathrm{u}\mathrm{r}\mathrm{c}\mathrm{e}\right)$ ≈ 0.07, which is 0.01 lower than the estimate given in Joachim et al. (2015). This results in an OIB source region fluorine concentration estimate of 30–70 ppm, which is about 10% lower than the estimate given in Joachim et al. (2015) that did not consider the effect of water. Consequently, results of this study show that water does affect the bulk fluorine partitioning behavior in OIB source regions and needs to be considered when estimating fluorine OIB source region concentrations. This includes the quantity of water present in the mantle as well as the amount of water that is introduced into sample charges during preparation and execution of nominally dry high-pressure–temperature experiments.

The estimate in this study does not consider the potential effect of water on the fluorine and chlorine partitioning behavior between pyroxenes or garnet and melt. A preliminary model presented by Dalou et al. (2014) considers the effect of water on the fluorine and chlorine partitioning behavior between opx, cpx, garnet, and melt, and predicts that the Cl/F ratio of melts increases with increasing fluid fraction, which may indicate that water affects the fluorine and/or chlorine partitioning behavior. Experiments of that study were, however, not only performed with varying melt water contents but also at different temperatures, which may strongly affect the partitioning behavior (Joachim et al. 2015). Results of other studies seem to indicate that there is no effect of water on the fluorine partitioning behavior between orthopyroxene and melt (Hauri et al. 2006), clinopyroxene and melt (O’Leary et al. 2010; Beyer et al. 2016), or garnet and melt (Hauri et al. 2006; Beyer et al. 2016) at Earth’s mantle conditions. Our study also does not consider a potential effect of iron (and *f*O_2_) on the partitioning behavior. Further studies are urgently required to determine the effect of such parameters. This will enable us to improve estimates of halogen abundances in MORB and OIB source regions and to better constrain recycling rates into and the distribution of volatiles within the Earth’s mantle.

### Potential effect of water on fluorine concentrations and F/H_2_O ratios during partial melting in the upper mantle

In a recent study, Beyer et al. (2016) discuss a potential process to generate magmas with a high F/H_2_O ratio in the upper mantle. In their model, bulk fluorine and water partition coefficients between mantle minerals and melt are kept constant regardless of potential effects of pressure, temperature, or bulk water content. Results of the model imply that multiple episodes of small degree melting are required to deplete a residual mantle more in H_2_O than in fluorine and thus obtain high F/H_2_O ratios between 0.1 and 0.9.

Results of this study show that the fluorine partitioning behavior between olivine and melt is affected by the bulk water content. Olivine is the most abundant mineral in peridotite with a modal abundance of 62% (McDonough 1990). This implies that variations in the fluorine partitioning behavior between olivine and melt may potentially play an important role in the generation of magmas containing a high fluorine concentration and a high F/H_2_O ratio.

Consider a scenario where small amounts of water (<1 wt% bulk water content) are added to a dry peridotitic system in the Earth’s upper mantle. This will decrease the solidus temperature and may lead to small degree partial melting. The model of Beyer et al. (2016) shows that the F/H_2_O ratio in the residual increases with increasing melt percentage, which is dependent on the amount of water that is added to the system. Simultaneously, addition of small amounts of water will increase the fluorine partition coefficient between olivine and melt (Fig. 3). Consequently, the addition of small amounts of water to a dry peridotitic system may lead to a significantly stronger increase in the F/H_2_O ratio of the residual during a small degree partial melting event than predicted by the model of Beyer et al. (2016) and may provide a mechanism that explains the generation of high F/H_2_O ratios and high fluorine concentrations in the upper mantle without the requirement of multiple partial melting episodes.

However, there are several assumptions in this scenario that need to be tested before it can be verified. We consider in this study only the bulk H_2_O content and not the water activity. Further experiments are required to investigate, if a decrease in water activity (e.g., through addition of CO_2_) affects the fluorine partitioning behavior between olivine and melt. Another assumption that needs to be tested is whether partitioning of water into olivine is affected by the addition of fluorine to the system. If the above-described incorporation mechanism of clumped OH/F defects in olivine ("Halogen incorporation mechanism in olivine") proposed by Crépisson et al. (2014) is correct, then it seems likely that OH partitioning into olivine is affected by fluorine as well, so that fluorine and water affect the partitioning behavior and thus the compatibility of each other. This would not only affect absolute water and fluorine concentrations in melts and residuals after partial melting events but also the respective F/H_2_O ratios.

Currently, the only conclusion that can be drawn with confidence is that the presence of small amounts of water needs to be considered when estimating the fluorine partition coefficient between olivine and silicate melt at Earth’s uppermost mantle conditions and the fluorine concentration in OIB source regions.

## Conclusions

- Results of this study show that there is within uncertainty no effect of water on the chlorine partitioning behavior between olivine and melt at 1280 °C, 0.3 GPa, and a bulk water content ranging from 0.03(2) to 0.33 (6) wt% H_2_O. In contrast, fluorine partition coefficients increase linearly within this range with
  $$D_{F}^{{{\text{ol/melt}}}} \; = \;3.6\; \pm \;0.4\;\; \times \;\;10^{{ - 3}} \;\; \times \;\;X_{{{\text{H}}_{{\text{2}}} {\text{O}}}} \left( {{\text{wt\% }}} \right)\; + \;6\; \pm \;0.4\;\; \times \;\;10^{{ - 4}} .$$
- The combined incorporation of fluorine and hydrogen into the olivine structure increases the partitioning of fluorine into olivine. This is consistent with the suggested formation of clumped OH/F defects in the forsterite structure (Crépisson et al. 2014), which increase the defect stability compared to solely hydrolytic weakening.
- The effect of water on the fluorine and chlorine partitioning behavior between olivine and melt can be neglected for estimates of chlorine in MORB and OIB source regions and fluorine in MORB source regions. In the simplified iron-free CMAS + F-Cl-Br-I-H_2_O system, only the effect of temperature on the partitioning behavior needs to be considered as a first-order controlling factor for the determination of these source region concentrations.
- Considering the effect of water on the fluorine partitioning behavior between olivine and melt indicates that fluorine OIB source region estimates are about 10% lower than previously expected (Joachim et al. 2015). This implies that the effect of water on the fluorine partitioning behavior between Earth’s mantle minerals and silicate melt needs to be considered for a correct estimation of fluorine abundances in OIB source regions.

## References

[CR1] Acosta-Vigil A, London D, Morgan GB VI, Dewers TA (2006). Dissolution of quartz, albite, and orthoclase in H_2_O-saturated haplogranitic melt at 800°C and 200MPa: diffusive transport properties of granitic melts at crustal anatectic conditions. J Pet.

[CR2] Berndt J, Liebske C, Holtz F, Freise M, Nowak M, Ziegenbein D, Hurkuck W, Koepke J (2002). A combined rapid-quench and H_2_-membrane setup for internally heated pressure vessels: description and application for water solubility in basaltic melts. Am Mineral.

[CR3] Bernini D (2011) Halogens and Trace Elements in Subduction Zones. Dissertation, University of Bayreuth

[CR4] Bernini D, Wiedenbeck M, Dolejs D, Keppler H (2013). Partitioning of halogens between mantle minerals and aqueous fluids: implications for the fluid flow regime in subduction zones. Contrib Mineral Petrol.

[CR5] Beyer C, Klemme S, Wiedenbeck M, Stracke A, Vollmer C (2012). Fluorine in nominally fluorine-free mantle minerals: experimental partitioning between olivine, orthopyroxene and silicate melts with implications for magmatic processes. Earth Planet Sci Lett.

[CR6] Beyer C, Klemme S, Grützner T, Ireland TR, Magee CW, Frost DJ (2016). Fluorine partitioning between eclogite garnet, clinopyroxene, and melt at upper mantle conditions. Chem Geol.

[CR7] Boettcher AL, Mysen BO, Allen JC (1973). Techniques for the control of water fugacity and oxygen fugacity for experimentation in solid-media high-pressure apparatus. J Geophys Res.

[CR8] Brodholt JP, Refson K (2000). An ab initio study of hydrogen in forsterite and a possible mechanism for hydrolytic weakening. J Geophys Res.

[CR9] Crépisson C, Blanchard M, Bureau H, Sanloup C, Withers AC, Khodja H, Surblé S, Raepsaet C, Béneut K, Leroy K, Giura P, Balan E (2014). Clumped fluoride-hydroxyl defects in forsterite: implications for the upper mantle. Earth Planet Sci Lett.

[CR10] Dalou C, Koga KT, Shimizu N, Boulon J, Devidal JL (2012). Experimental determination of F and Cl partitioning between lherzolite and basaltic melt. Contrib Mineral Petrol.

[CR11] Dalou C, Koga KT, Le Voyer M, Shimizu N (2014). Contrasting partitioning behavior of F and Cl during hydrous mantle melting: implications for F/Cl signature in arc magmas. Prog Earth Planet Sci.

[CR12] Danyushevsky LV, Eggins SM, Fallon TJ, Christie DM (2000). H_2_O abundance in depleted to moderately enriched mid-ocean ridge magmas. Part I: incompatible behavior, implications for mantle storage, and origin of regional variations. J Petrol.

[CR13] Déruelle B, Dreibus G, Jambon A (1992). Iodine abundances in oceanic basalts: implications for Earth dynamics. Earth Planet Sci Lett.

[CR14] Dixon JE, Clague DA, Wallace P, Poreda R (1997). Volatiles in alkali basalts from the North Arch Volcanic Field, Hawaii: extensive degassing of deep submarine-erupted alkali series lavas. J Petrol.

[CR15] Dixon JE, Leist L, Langmuir C, Schilling JG (2002). Recycled dehydrated lithosphere observed in plume-influenced mid-ocean-ridge basalt. Nature.

[CR16] Drury MR (1991). Hydration-induced climb dissociation of dislocations in naturally deformed mantle olivine. Phys Chem Miner.

[CR17] Erdmann M, Koepke J (2016). Experimental temperature cycling as a powerful toll to enlarge melt pools and crystals at magma storage conditions. Am Mineral.

[CR18] Green DH, Hibberson WO, Rosenthal A, Kovács I, Yaxley GM, Fallon TJ, Brink F (2014). Experimental study of the influence of water on melting and phase assemblages in the upper mantle. J Petrol.

[CR19] Hauri EH (2002). SIMS analysis of volatiles in silicate glasses, 2: Isotopes and abundances in Hawaiian melt inclusions. Chem Geol.

[CR20] Hauri EH, Gaetani GA, Green TH (2006). Partitioning of water during melting of the Earth’s upper mantle at H_2_O-undersaturated conditions. Earth Planet Sci Lett.

[CR21] Hermann J, Fitz Gerald J, Malaspina N, Berry AJ, Scambelluri M (2007). OH-bearing planar defects in olivine produced by the breakdown of Ti-rich humite minerals from Dabie Shan (China). Contrib Mineral Petrol.

[CR22] Hirschmann MM (2006). Water, melting, and the deep Earth H_2_O cycle. Annu Rev earth Planet Sci.

[CR23] Hunt JB, Hill GP (1993). Tephra geochemistry: a discussion of some persistent analytical problems. The Holocene.

[CR24] Ito E, Harris DM, Anderson AT (1983). Alteration of oceanic crust and geologic cycling of chlorine and water. Geochim Cosmochim Acta.

[CR25] Jambon A, Déruelle B, Dreibus G, Pineau F (1995). Chlorine and bromine abundance in MORB: the contrasting behavior of the Mid-Atlantic Ridge and East Pacific Rise and implictaions for chlorine geodynamic cycle. Chem Geol.

[CR26] Jamtveit B, Brooker R, Brooks K, Larsen LM, Pedersen T (2001). The water content of olivines from the North Atlantic Volcanic Province. Earth Planet Sci Lett.

[CR27] Joachim B, Gardés E, Velickov B, Abart R, Heinrich W (2012). Experimental growth of diopside + merwinite reaction rims: The effect of water on microstructure development. Am Mineral.

[CR28] Joachim B, Pawley A, Lyon IC, Marquardt K, Henkel T, Clay PL, Ruzié L, Burgess R, Ballentine CJ (2015). Experimental partitioning of F and Cl between olivine, orthopyroxene and silicate melt at Earth’s mantle conditions. Chem Geol.

[CR29] Johnson L, Burgess R, Turner G, Milledge JH, Harris JW (2000). Noble gas and halogen geochemistry of mantle fluids: comparison of African and Canadian diamonds. Geochim Cosmochim Acta.

[CR30] Kitamura M, Kondoh S, Morimoto N, Miller GH, Rossman GR, Putnis A (1987). Planar OH-bearing defects in mantle olivine. Nature.

[CR31] Konács I, Green DH, Rosenthal A, Hermann J, O’Neill HSt, Hibberson WO, Udvardi B (2012). An experimental study of water in nominally anhydrous minerals in the upper mantle near water-saturated solidus. J Petrol.

[CR32] Kovalenko MA, Naumov V, Girnis A, Dorofeeva V, Yarmolyuk V (2006). Composition and chemical structure of oceanic mantle plumes. Petrology.

[CR33] Kuehn S, Froese D, Pearce N, Foit F (2009) ID3506, a new/old Lipari obsidian standard for characterization of natural glasses and for tephrochronology. AGU fall meeting 2009, V31E-2010 (abstr.)

[CR34] Le Roux PJ, Shirey SB, Hauri EH, Perfit MR, Bender JF (2006). the effects of variable sources, processes and contaminants on the composition of the northern EPR MORB (8–10°N and 12–14°N): evidence from volatiles (H_2_O, CO_2_, S) and halogens (F, Cl). Earth Planet Sci Lett.

[CR35] Ludwig T, Stalder R (2007). A new method to eliminate the influence of in situ contamination in SIMS analysis of hydrogen. J Anal At Spectrom.

[CR36] Matjuschkin V, Brooker RA, Tattich B, Blundy JD, Stamper CC (2015). Control and monitoring of oxygen fugacity in piston cylinder experiments. Contrib Mineral Petrol.

[CR73] McDonough WF (1990). Constraints on the composition of the continental lithospheric mantle. Earth Planet Sci Lett.

[CR37] McDonough WF, Sun SS (1995). The composition of the Earth. Chem Geol.

[CR38] Mercier M, Di Muro A, Métrich N, Giordano D, Belhadj O, Mandeville CW (2010). Spectroscopic analysis (FTIR, Raman) of water in mafic and intermediate glasses and glass inclusions. Geochim Cormochim Acta.

[CR39] Michael PJ (1988). The concentration, behaviour and storage of H_2_O in the suboceanic upper mantle: implications for mantle metasomatism. Geochim Cosmochim Acta.

[CR40] Michael PJ (1995). Regionally distinctive sources of depleted MORB: evidence from trace elements and H_2_O. Earth Planet Sci Lett.

[CR41] Michael PJ, Schilling JG (1989). Chlorine in mid-ocean ridge magmas: evidence for assimilation of seawater-influenced components. Geochim Cosmochim Acta.

[CR42] Newsom HE (1995) Composition of the solar system, planets, meteorites, and major terrestrial reservois. In: Global Earth Physics, A Handbook of Physical Constants, AGU Reference Shelf, 3rd edn. Elsevier, pp 123–166

[CR43] Nichols ARL, Carroll MR, Höskuldsson A (2002). Is the Iceland hot spot also wet? Evidence from the water contents of undegassed submarine and subglacial pillow basalt. Eart Planet Sci Lett.

[CR44] O’Leary J, Gaetani GA, Hauri EH (2010). The effect of tetrahedral Al^3+^ on the partitioning behavior of water between clinopyroxene and silicate melt. Earth Planet Sci Let.

[CR45] Palme H, O’Neill HSC (2014) Cosmochemical estimates of mantle composition. In: Holland HD, Turekian KK (eds) Treat Geochem, vol. 3. Elsevier Ltd, pp 1–39

[CR46] Patiño Douce AE, Beard JS (1994). H_2_O loss from hydrous melts during fluid-absent piston cylinder experiments. Am Mineral.

[CR47] Pyle DM, Mather TA (2009). Halogens in igneous processes and their fluxes to the atmosphere and oceans from volcanic activity: a review. Chem Geol.

[CR48] Risold AC, Trommsdrof V, Grobéty B (2001). genesis of ilmenite rods and palisades along himite-type defects in olivine from Alpe Arami. Contrib Mineral Petrol.

[CR49] Rosenthal A, Hauri EH, Hirschmann MM (2015). Experimental determination of C, F, and H partitioning between mantle minerals and carbonatzed basalt, CO_2_/Ba and CO_2_/Nb systematics of partial melting, and the CO_2_ contents of basaltic source regions. Earth Planet Sci Lett.

[CR50] Ruzié-Hamilton L, Clay PL, Burgess R, Joachim B, Ballentine CJ, Turner G (2015). Determination of halogen abundances in terrestrial and extraterrestrial samples by the analysis of noble gases produced by neutron irradiation. Chem Geol.

[CR51] Saal AE, Hauri EH, Langmuir CH, Perfit MR (2002). Vapour undersaturation in primitive mid-ocean-ridge basalt and the volatile content of the Earth’s upper mantle. Nature.

[CR52] Salters VJM, Stracke A (2004) Composition of the depleted mantle. Geochem Geophys Geosyst 5 (art. No. Q05004)

[CR53] Schilling JG, Bergeron MB, Evans R (1980). Halogens in the mantle beneath the North-Atlantic. Philos Trans R Soc Lond Ser A-Math Phys Eng Sci.

[CR54] Seaman C, Sherman SB, Garcia MO, Baker MB, Balta B, Stolper E (2004). Volatiles in glasses from the HSDP2 drill core. Geochem Geophys Geosyst.

[CR55] Shaw DM (1970). Trace element fractionation during anataxis. Geochim Cosmochim Acta.

[CR56] Shaw AM, Hauri EH, Fischer TP, Hilton DR, Kelley KA (2008). Hydrogen isotopes in Mariana arc melt inclusions: implications for subduction and dehydration and the deep-Earth water cycle. Earth Planet Sci Lett.

[CR57] Simons K, Dixon J, Schilling JG, Kingsley R, Poreda R (2002). Volatiles in basaltic glasses from the Easter-Salas y Gomez seamount chain and Easter microplate: implications for geochemical cycling of volatile elements. Geochem Geophys Geosyst.

[CR58] Smyth JR, Mardel RE, McCormick TC, Monuz JL, Rossman GR (1990). Crystal structure refinement of a F-bearing spessartine garnet. Am Mineral.

[CR59] Smyth JR, Frost DJ, Nestola F, Holl CM, Bromiley G (2006). olivine hydration in the deep upper mantle: effect of temperature and silica activity. Geophys Res Lett.

[CR60] Stalder R, Ulmer P (2001). Phase relations of a serpentine composition between 5 and 14 GPa: significance of clinohumite and phase E as water carriers into the transition zone. Contrib Mineral Petrol.

[CR61] Steinbach V, Yuen DA (1995). The effects of temperature-dependent viscosity on mantle convection with the two major phase transitions. Phys Earth Planet Inter.

[CR62] Stephan T, Lyon IC (2013) Applications of ToF-SIMS in cosmochemistry. In: Vickerman JC, Briggs D (eds), ToF-SIMS: materials analysis by mass spetrometry, 2nd ed. IM publications LLP and SurfaceSpectra Limited

[CR63] Straub SM, Layne GD (2003). The systematics of chlorine, fluorine, and water in Izu arc front volcanic rocks: implications for volatile recycling in subduction zones. Geochim Cosmochim Acta.

[CR64] Truckenbrodt J, Johannes W (1999). H_2_O loss during piston-cylinder experiments. Am Mineral.

[CR65] Valley JW, Essene EJ, Peacor DR (1983). Fluorine-bearing garnets in Adirondack calc-silicates. Am Mineral.

[CR66] Visser D (1993). Fluorine-bearing hydrogarnets from Blengsvatn, Bamble sector, south Norway. Mineral Petrol.

[CR68] Wallace PJ, Frey FA, Weis D, Coffin MF (2002). Origin and evolution of the Kerguelen Plateau, Broken Ridge and Kerguelen Archipelago: editorial. J Petrol.

[CR69] Wedepohl JD (1995). The composition of the continental crust. Geochim Cosmochim Acta.

[CR70] Wirth R, Dobrzhinetskaya LF, Green HW (2001). Electron microscope study of the reaction olivine + H_2_O → titanian clinohumite + titanian chondrodite synthesized at 8 GPa, 1300 K. Am Mineral.

[CR71] Workman RK, Hauri EH, Hart SR, Wang J, Blusztajn J (2006). Volatile and trace elements in basaltic glasses from Samoa: implications for water distribution in the mantle. Earth Planet Sci Lett.

[CR72] Xue X, Kanzaki M, Turner D, Loroch D (2016) Hydrogen incorporation mechanisms in forsterite: ^1^H NMR measurement and first principles calculation. Goldschmidt Abstracts, 2016 3503

